# Efficacy and Safety of Respiratory Telerehabilitation in Patients with Long COVID-19: A Systematic Review and Meta-Analysis

**DOI:** 10.3390/healthcare11182519

**Published:** 2023-09-12

**Authors:** Andrés Calvache-Mateo, Alejandro Heredia-Ciuró, Javier Martín-Núñez, Sofía Hernández-Hernández, Gregory Reychler, Laura López-López, Marie Carmen Valenza

**Affiliations:** 1 Department of Physiotherapy, Faculty of Health Sciences, University of Granada, Av. De la Ilustración, 60, 18016 Granada, Spain; andrescalvache@ugr.es (A.C.-M.); ahc@ugr.es (A.H.-C.); javimn@ugr.es (J.M.-N.); shernandez@correo.ugr.es (S.H.-H.); cvalenza@ugr.es (M.C.V.); 2 Institut de Recherche Expérimentale et Clinique (IREC), Pôle de Pneumologie, ORL & Dermatologie, Université Catholique de Louvain, 1200 Bruxelles, Belgium; gregory.reychler@saintluc.uclouvain.be; 3 Service de Médecine Physique et Réadaptation, Cliniques Universitaires Saint-Luc, 1200 Bruxelles, Belgium; 4 Service de Pneumologie, Cliniques Universitaires Saint-Luc, 1200 Bruxelles, Belgium

**Keywords:** Long COVID-19, telerehabilitation, quality of life, dyspnea, adverse effects, functional capacity

## Abstract

The aim of this review was to identify, map, and synthesize the extent and nature of research activity on the use of telerehabilitation to support Long COVID-19 rehabilitation and examine the efficacy and safety of respiratory telerehabilitation in patients with Long COVID-19. A systematic review and meta-analysis of randomized controlled trials were performed. We included controlled trials that tested the effect of respiratory telerehabilitation interventions in patients with Long COVID-19 versus no intervention, usual care, placebo, or face-to-face intervention. The data were pooled, and a meta-analysis was completed for quality of life, dyspnea, lung function, anxiety and depression, respiratory muscle strength, functional capacity, and lower limb strength. Finally, 10 studies were included. The meta-analysis results show significant differences in favor of respiratory telerehabilitation in quality of life (*p* = 0.02), dyspnea (*p* < 0.00001), respiratory muscle strength (*p* < 0.001), functional capacity (*p* < 0.0001), and lower limb strength (*p* = 0.01) but not in lung function (*p* = 0.28) and anxiety and depression (*p* = 0.55). In addition, there were no statistically significant differences in adverse effects (*p* = 0.06) between the telerehabilitation and comparator groups. The results suggest that these interventions can improve quality of life, reduce dyspnea, and increase respiratory and lower extremity muscle strength as well as functional capacity in patients with Long COVID-19.

## 1. Introduction

Long COVID-19, defined by the WHO as the onset of COVID-19 with symptoms that last after infection for at least 2 months and cannot be explained by an alternative diagnosis [1,2,3], affects between 5.4 and 17.9 million people worldwide and is one of the leading causes of disability [4].

The estimated prevalence of Long COVID-19 is approximately 23% of people with at least one post-COVID condition, specifically 35% in patients treated for COVID-19 on an outpatient basis but approximately 87% among cohorts of hospitalized patients [5,6]. In this line, these symptoms are leading contributors to the rapid increase in the demand for health services worldwide over the last few years [7,8] with most of the expenditure increase occurring in pain, respiratory difficulties, hyperlipidemia, malaise and fatigue, and hypertension [9,10,11]. Given their significant and growing financial burden [7,8], potential efficiencies in the model of care for patients with Long COVID-19 are a matter of considerable policy interest.

Traditionally, rehabilitation services form a core component of the care pathway for any disabled patient as a means of facilitating the recovery of functional independence in rehabilitation centers or hospitals. Additionally, the increased life expectancy and the limited resources in public health highlight the importance of reaching effective and sustainable rehabilitation services to cope with the needs of the population [12].

Recent advances in digital and telecommunication technologies, such as e-Health, telemedicine, wearable devices, virtual reality, and online educational tools, have made healthcare services more affordable and convenient for consumers [13]. The pandemic and the situation generated by COVID-19 have intensified the use of telerehabilitation in the healthcare sector. There is an increasing number of studies that have proven the efficacy of telerehabilitation in other pathologies, although on occasions, it has been difficult to establish generalizations due to the heterogeneity of the interventions [14]. This has created an unprecedented opportunity for the rehabilitation of patients with Long COVID-19 to adapt to new approaches to care by using innovations in digital technology.

Telerehabilitation is emerging as a viable substitute for in-person rehabilitation, particularly in the realm of cardiac and pulmonary rehabilitation, among other areas [15]. Both physical and cognitive virtual reality exercises administered through telerehabilitation have demonstrated their effectiveness and safety in addressing post-COVID-19 conditions in patients [16]. Patient satisfaction, as reported in various studies conducted during and after periods of confinement, underscores the significance of incorporating telerehabilitation into the comprehensive rehabilitation of individuals in the acute phase and those experiencing COVID-19-related sequelae [17].

A growing body of literature supports the use of telerehabilitation to improve patient satisfaction and health outcomes for a diverse range of clinical conditions, such as neurological diseases [18,19], stroke [20], cancer [21], and cardiac and pulmonary rehabilitation [22]. The British Society of Rehabilitation Medicine [23], Chartered Society of Physiotherapy [24], and the British Thoracic Society [25] have each issued policy documents regarding COVID-19 rehabilitation. Nonetheless, there is still a scarcity of concrete evidence regarding the most effective approach to deliver rehabilitation in this particular context. While the ideal rehabilitation strategy for COVID-19 remains uncertain, three fundamental components are relevant to the rehabilitation of nearly all conditions: (1) exercise training; (2) education, which includes self-management; and (3) psychosocial support [26].

However, it has not yet been demonstrated for Long COVID-19. Furthermore, it cannot be assumed that all patients with Long COVID-19 can safely be involved in telerehabilitation, considering the heterogeneity of prognostic outcomes. Nevertheless, the up-to-date evidence base about the use of telerehabilitation for Long COVID-19 rehabilitation has not been reviewed and mapped. Therefore, the aim of this review was to identify, map, and synthesize the extent and nature of research activity on the use of telerehabilitation to support Long COVID-19 rehabilitation. Additionally, the literature surrounding the safety of telerehabilitation interventions in patients with Long COVID-19 has not been specifically reviewed.

## 2. Methods

### 2.1. Protocol and Registration

A systematic review and meta-analysis were performed in accordance with the principles outlined in the Preferred Reporting Items for Systematic Reviews and Meta-Analyses (PRISMA) statement guidelines [27] and the Cochrane Collaboration guidelines for evaluating interventions [28]. The protocol for this systematic review was duly registered on PROSPERO (registration number: CRD42022373781, registration date: 7 November 2022).

### 2.2. Search Strategy

We conducted a systematic search of articles indexed on MEDLINE (via PubMed), Scopus, and PEDro that covered the period from the inception of the databases until July 2023. The screening and analysis of the studies took place between November 2022 and July 2023. We developed a search strategy in MEDLINE using the following steps: (1) the development of keywords by examining relevant key terms used in the existing systematic reviews, (2) examination of the MeSH database, and (3) expert guidance and review by a specialist.

The search strategy was rigorously tested and refined to ensure its effectiveness for this review. Subsequently, the strategy was adapted to accommodate the differences in indexing across the other databases (Appendix A). To supplement our search, we manually checked the reference lists of the included studies and relevant review articles to identify any additional articles not captured in the systematic review of the databases.

To formulate the research question, we applied the PICOS model (Participants, Interventions, Comparisons, Outcome, and Study Design).

P (Participants): adults with Long COVID-19 syndrome without restrictions on gender, ethnicity, and setting.

I (Intervention): respiratory telerehabilitation interventions.

C (Comparison): the respiratory telerehabilitation had to be compared to no intervention, usual care, placebo, or face-to-face intervention.

O (Outcomes): quality of life, symptoms, physical capacity, function, and psychological well-being.

S (Study Design): randomized clinical trials were included.

Only full-text, randomized controlled trials written in English, Spanish, and French were included in the systematic review. Systematic reviews and meta-analyses, observational studies, clinical practice guidelines, letters, abstracts, editorials, conference papers, theses, and dissertations were excluded.

In this line, respiratory telerehabilitation interventions were considered as any intervention with the ability to provide distance support, evaluation, and intervention to persons who are disabled via telecommunication [29,30].

Once the records were retrieved from the various databases, duplicate entries were removed to ensure data accuracy. Subsequently, two reviewers (A.C and C.V) conducted separate evaluations of the titles and abstracts of all the articles to assess their relevance for potential inclusion. The studies that met the eligibility criteria were further scrutinized in detail. In case of any discrepancies or disagreements between the two reviewers, a third reviewer (G.R) was asked to resolve the differences and arrive at a consensus on the final selection of studies.

After the article’s selection and the data extraction, we performed a methodological quality assessment with the Downs and Black quality assessment method [31]. This method has 27 items that comprise five subscales (study quality, external validity, study bias, confounding and selection bias, and study power), classifying methodological quality as “excellent” if studies have a 26 or higher score, between 20 and 25 “good”, between 15 and 19 “fair”, and 14 or lower “poor”. This scale is ranked as one of the six highest-quality assessment scales suitable for use in systematic reviews due to the high validity and reliability presented [32,33].

The risk of bias for the included randomized controlled trials was assessed with the Cochrane Risk-of-Bias tool version 2.0 (RoB-2) [34]. This tool consists of five domains that focus on the randomization process, deviations from the intended interventions, missing outcome data, measurement of the outcome, and the selection of the reported result. The studies were interpreted as having a high, low, or unclear risk of bias.

### 2.3. Meta-Analysis

The study results with respect to quality of life, dyspnea, lung function, anxiety and depression, respiratory muscular strength, functional capacity, lower limb strength, and adverse events were pooled, and a meta-analysis was undertaken using Review Manager software (Rev-Man version 5.1, updated March 2011) and the Cochrane Collaboration guidelines for reviewing interventions [35].

Post-intervention means and standard deviations were utilized as the primary data for pooling the results. In cases where the data were insufficient for the meta-analysis (e.g., missing means or standard deviations), efforts were made to contact the authors of the respective trials to obtain the required information. In instances where standard deviations were not provided but *p*-values or 95% confidence intervals were available, these were used to calculate the missing standard deviations using the embedded Review Manager calculator. Additionally, if a trial compared multiple intervention arms, each arm was treated as a separate entity in the meta-analysis, allowing for a comprehensive and accurate analysis of the data. These procedures helped ensure that the meta-analysis was conducted with the most complete and accurate data available, maximizing the reliability and validity of the findings.

Continuous outcomes were analyzed using weighted mean differences when all studies measured outcomes on the same scale. Standardized mean differences were used when all scales were assumed to measure the same underlying symptom or condition, but some studies measured the outcomes on different scales. The 95% confidence intervals were computed for all outcomes.

Finally, a meta-analysis of the adverse effects that may be generated with respiratory telerehabilitation using OR (odds ratio) was performed. When performing the meta-analysis of adverse effects using OR, since some of the treatment or comparator groups had no adverse effects, the continuity correction technique was used [36]. The continuity correction is a strategy applied to avoid mathematical or statistical problems when the cells of a contingency table (used to calculate the OR) have small or null values. In these cases, a constant value (e.g., 1) is usually added to all the cells of the table to ensure that there are no null values and that the calculations are valid.

The overall mean effect sizes were estimated using random-effect models or fixed-effect models according to the statistical heterogeneity I^2^ tests. I^2^ > 50% is considered to be a heterogeneous meta-analysis, and a random-effects model was used. A visual inspection of the forest plots for outlier studies was also undertaken. Sources of heterogeneity were explored, and sensitivity analyses were conducted by excluding trials that were at a high risk of detection or attrition bias.

A sensitivity analysis was conducted to investigate potential sources of heterogeneity and to determine how sensitive the conclusions of the study are to the particular method or study design feature that was used. If the effect and confidence intervals in the sensitivity analysis lead to the same conclusion as the primary meta-analysis value, the results are deemed robust.

## 3. Results

### 3.1. Study Selection

**Figure 1:**
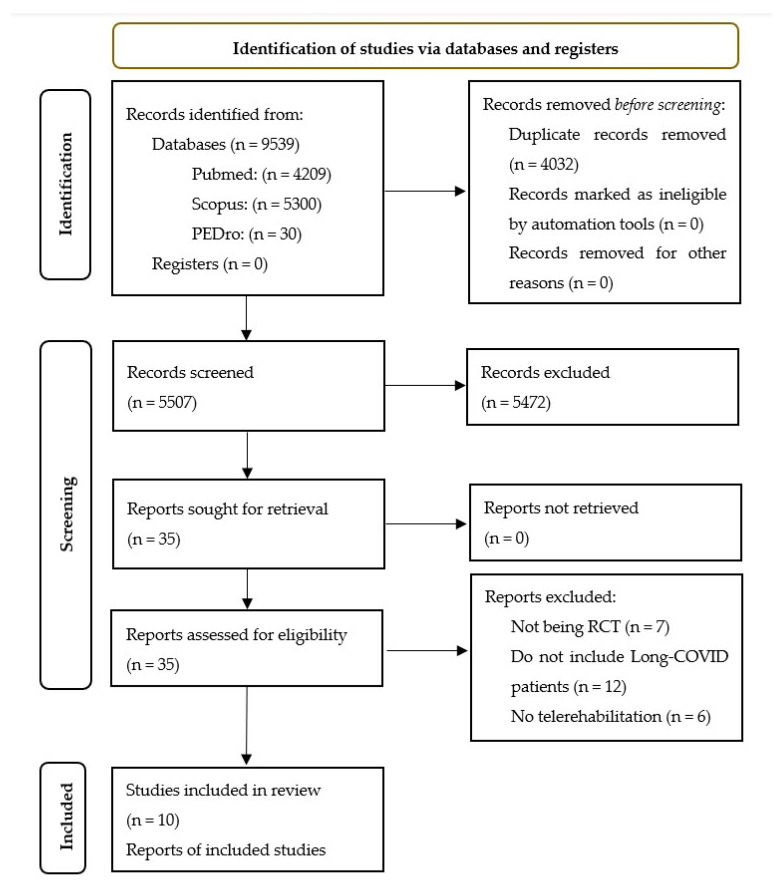
Flow diagram of the included articles.

A comprehensive search was conducted in the selected databases that found a total of 9539 records. After removing duplicates, 5507 records remained, which were screened based on their title and abstract. Only 35 articles were selected for full-text evaluation. After reviewing the inclusion and exclusion criteria, 10 studies were deemed eligible for inclusion in the qualitative and quantitative syntheses [37,38,39,40,41,42,43,44,45,46] (Figure 1).

### 3.2. Study Characteristics

The characteristics of the sample and the methodological evaluation of the included studies are shown in Table 1. A total of 866 patients with Long COVID-19 were included in the systematic review with a female predominance (73%) and an age range from 40 to 55 years.

Among the included articles, four studies [38,39,41,46] featured patients who had experienced acute COVID-19 infection more than 9 months prior, while four articles [37,40,43,44] focused on patients who had been infected 3 months prior. Two of the articles did not report the time elapsed since acute infection [42,45].

This systematic review examined the hospitalization status of patients across the included studies. Two articles exclusively studied patients who had been hospitalized [37,40], indicating severe or critical acute infection severity, while two other articles only included patients with mild acute infection severity who did not require hospitalization [42,45]. The remaining studies encompassed patients with a spectrum of severities [38,39,41,43,46] from mild to critical with a lower percentage of patients requiring hospitalization compared to the hospitalized groups [38,41,44]. Three articles did not report on the hospitalization status of patients [39,42,46]. Regarding the duration of hospitalization, only the articles that exclusively studied hospitalized patients [37,40] reported on the days of hospitalization, which ranged from 9.5 to 26.18 days.

**Figure 2:**
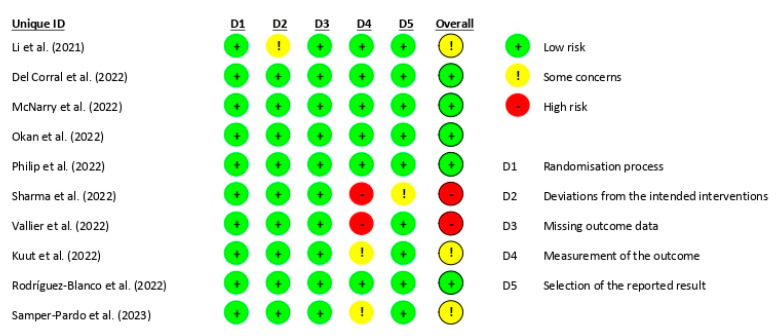
Risk of bias [37,38,39,40,41,42,43,44,45,46].

This systematic review evaluated the methodological quality of the studies using the Downs and Black quality assessment method. Of the studies included, one was classified as excellent [38], while seven were classified as good [37,39,40,41,44,45,46], one was classified as fair [43], and one was classified as poor [42]. Additionally, the risk of bias of all ten studies [37,38,39,40,41,42,43,44,45,46] was assessed using the RoB-2 tool (Figure 2), which concluded that five of the articles had a low risk of bias [38,39,40,41,45], three of them had some concerns [37,44,46], and two others had a high risk of bias [42,43]. These findings suggest that most of the studies included in this review were conducted with rigorous methodology.

The details of the interventions performed and the results obtained are shown in Table 2. The modality of the respiratory telerehabilitation performed varied among the different studies included. Therefore, eight of the included studies performed respiratory training or breathing exercises [37,38,39,40,41,42,45,46]. Four of them combined it with aerobic exercise or strength training [37,42,45,46]. Philip et al. [41] combined breathing exercises with anxiety self-management exercises, and the rest performed the breathing exercises or respiratory training in isolation [38,39,40]. Kuut et al. [44] conducted a multidisciplinary telerehabilitation program based on cognitive behavioral therapy. Vallier et al. [43] conducted a telerehabilitation program that combined aerobic exercises, strength exercises, and relaxation exercises and compared it with the same rehabilitation program but applied in a face-to-face setting. For this reason, it was included in the qualitative analysis but not in the quantitative analysis.

With respect to the different components of the telerehabilitation programs, the most repeated were tele-education in self-management, symptom and mood telemonitoring, physical activity telemonitoring with personalized feedback, and teleconsultation with healthcare professionals that were included in up to eight of the ten studies. Remote decision-support systems and telecommunication with other patients were included in four of the ten studies.

The duration of the respiratory telerehabilitation programs ranged from 4 weeks to 17 weeks with the most repeated duration being 6 weeks of treatment. The duration in minutes of the different respiratory telerehabilitation sessions ranged between 20 and 60, and they were performed 3 to 7 days a week, repeating two times a day in the study of Del Corral et al. [38] and three times a day in the study of Okan et al. [40]. All the articles included in this review adjusted the volume of the interventions with continuous reevaluations.

The intensity of the respiratory telerehabilitation was regulated according to the maximal inspiratory pressure (MIP) [38], sustained maximal inspiratory pressure (SMIP) [39], or heart rate (HR) [43] values in three of the articles; in five articles, it was adjusted to each participant based on continuous feedback [37,41,44,45,46]; and two articles [40,42] did not specify the way to regulate the intensity of the respiratory training.

The interventions included in this review were all home-based, and most were monitored. The most common form of monitoring was weekly, daily, or on-demand online sessions, which could be group or individual. Three articles did not monitor the interventions in any way [39,42,46]. The technology most commonly used by the different authors to conduct the telerehabilitation interventions was videoconferencing followed by mobile apps.

With respect to the comparison of interventions, five of the studies compared respiratory telerehabilitation with the usual care [39,41,42,44,46], and two other articles compared respiratory telerehabilitation against an educational brochure [37,40]. Del Corral et al. [38] compared their respiratory training with a sham respiratory training with the same treatment but performed with valveless devices that did not oppose resistance to the patient. Finally, Rodriguez-Blanco et al. [45] compared their intervention with no intervention, and Vallier et al. [43] compared their intervention with the same intervention carried out in person.

### 3.3. Results Obtained in Meta-Analysis

**Figure 3:**
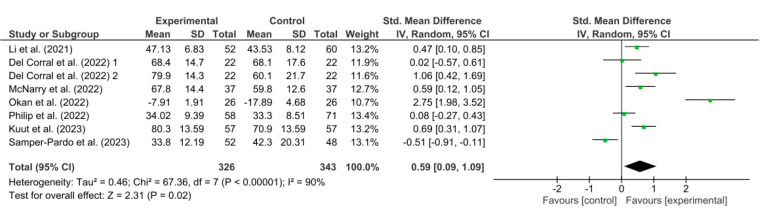
Results of the quality of life [37,38,39,40,41,44,46].

The results obtained in the meta-analysis with respect to quality of life were analyzed as shown in Figure 3. The pooled standardized mean difference (SMD) showed a significant overall effect of respiratory telerehabilitation compared to the comparator groups (SMD = 0.59; 95% CI = 0.09; 1.09; *p* = 0.02). The results showed heterogeneity, detecting a significant variability of I2 = 90%, not attributable to chance.

**Figure 4:**
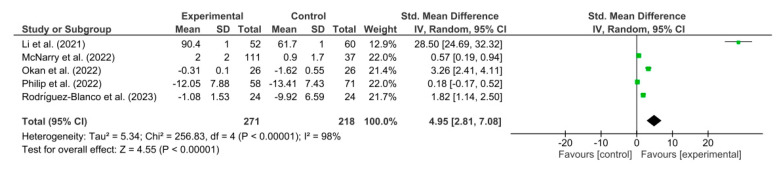
Results of dyspnea [37,39,40,41,45].

Figure 4 shows the results obtained in the meta-analysis for dyspnea. The pooled standardized mean difference (SMD) showed a significant overall effect of respiratory telerehabilitation compared to the comparator groups (SMD = 4.95; 95% CI = 2.81; 7.08; *p* < 0.00001). The results show heterogeneity, detecting a significant variability of I2 = 98%, not attributable to chance.

**Figure 5:**
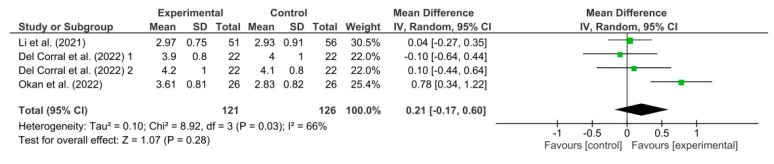
Results of FVC [37,38,40].

Figure 5 shows the results obtained in the meta-analysis for FVC. The pooled mean difference (MD) showed a non-significant overall effect of respiratory telerehabilitation compared to the comparator groups (MD = 0.21; 95% CI = −0.17; 0.60; *p* = 0.28). The results show heterogeneity, detecting a significant variability of I2 = 66%, not attributable to chance.

**Figure 6:**
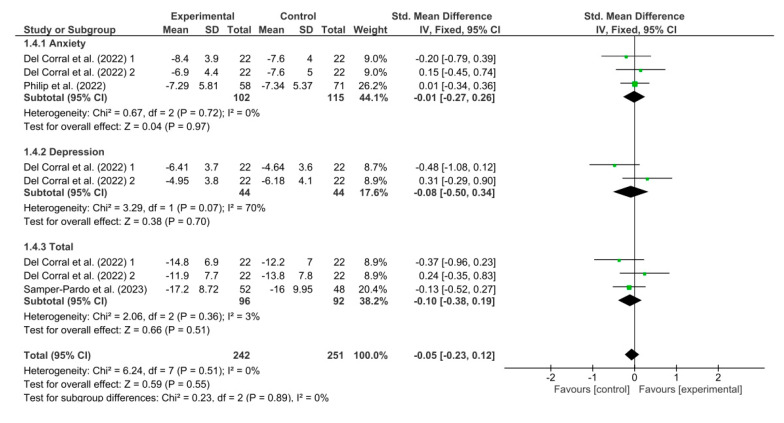
Results of anxiety and depression [38,41,46].

Figure 6 shows the results obtained in the meta-analysis for anxiety and depression. The pooled standardized mean difference (SMD) showed a non-significant overall effect of respiratory telerehabilitation compared to the comparator groups (SDM = −0.05; 95% CI = −0.23; 0.12; *p* = 0.55). The results do not show heterogeneity, detecting a significant variability of I2 = 0%.

**Figure 7:**
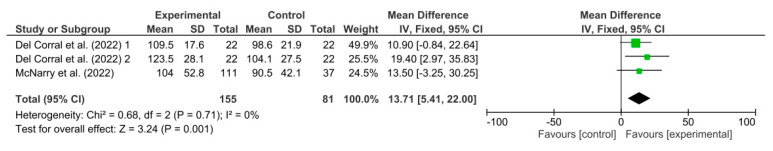
Results of MIP [38,39].

Figure 7 shows the results obtained in the meta-analysis for respiratory muscular strength. The pooled mean difference (MD) showed a significant overall effect of respiratory telerehabilitation compared to the comparator groups (MD = 13.71; 95% CI = 5.41; 22; *p* < 0.001). The results do not show heterogeneity, detecting a significant variability of I2 = 0%.

**Figure 8:**
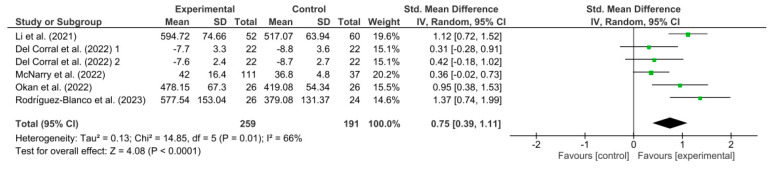
Results of functional capacity [37,38,39,40,45].

Figure 8 shows the results obtained in the meta-analysis for functional capacity. The pooled standardized mean difference (SMD) showed a significant overall effect of respiratory telerehabilitation compared to the comparator groups (SMD = 0.75; 95% CI = 0.39; 1.11; *p* < 0.0001). The results show heterogeneity, detecting a significant variability of I2 = 66%, not attributable to chance.

**Figure 9:**
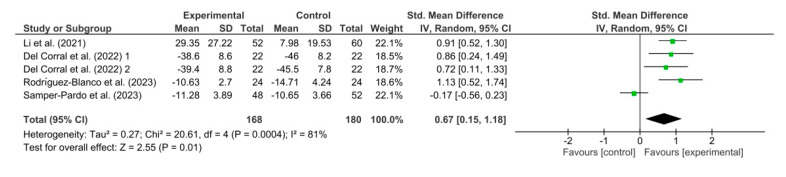
Results of lower limb strength [37,38,45,46].

Figure 9 shows the results obtained in the meta-analysis for lower limb strength. The pooled standardized mean difference (SMD) showed a significant overall effect of respiratory telerehabilitation compared to the comparator groups (SMD = 0.67; 95% CI = 0.15; 1.18; *p* = 0.01). The results show heterogeneity, detecting a significant variability of I2 = 81%, not attributable to chance.

**Figure 10:**
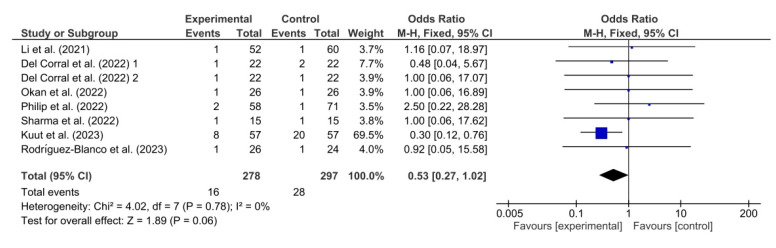
Results of adverse events [37,38,40,41,42,44,45].

Figure 10 shows the results obtained in the meta-analysis for adverse events. The result shown by this meta-analysis (OR = 0.53; 95% CI = 0.27; 1.02; *p* = 0.06) means that the difference between the groups exposed to telerehabilitation and the comparison groups is not statistically significant, implying that there is no strong evidence of an association between telerehabilitation and adverse effects.

## 4. Discussion

The COVID-19 pandemic has had a significant impact on the health and well-being of the worldwide population. One of the most worrying consequences of this disease is the syndrome known as Long COVID-19 where symptoms persist after acute infection and affect patients’ quality of life. This systematic review and meta-analysis aimed to evaluate the efficacy and safety of respiratory telerehabilitation as a potential intervention for the management of persistent Long COVID-19 symptoms.

The sample of our systematic review shows the characteristics of the Long COVID-19 population. The articles analyzed included only patients with Long COVID-19; however, the severity of the patients’ symptoms during the acute phase was variable. Of the 866 patients with Long COVID-19, 73.32% were women, and the age ranged from 40 to 55 years; the data are consistent with the characteristics of this population since it has been shown to be a disease more prevalent in women [47,48,49].

The respiratory telerehabilitation programs were heterogeneous among themselves with the most repeated parameters being 6 weeks of treatment at least three times a week with a duration of 20 to 60 min per session. These parameters are in line with the parameters of different pulmonary telerehabilitation programs presented in a review carried out in patients with COVID-19 [50]. Due to the pandemic situation in which the studies included in this review were carried out, all the interventions were performed at home [29,30,31,32,33,34,35,36,37,38], and most of them were monitored [37,38,40,41,43,44,45].

With respect to the components included within the telerehabilitation programs, the results found in this systematic review are in line with previous reviews in which it was shown that the most common and promising interventions are based on a combination of self-management tele-education, telemonitoring of symptoms and mood, telemonitoring of physical activity with personalized feedback, and teleconsultation with healthcare professionals [51,52].

The results of this review highlight the growing interest in the application of telerehabilitation in the management of patients with Long COVID-19. Ten studies were identified that met the inclusion criteria, suggesting that this area of research is of increasing interest. Most of the studies obtained a rating of good or excellent methodological quality, which increases the confidence in the results obtained.

The results of this review indicate that respiratory telerehabilitation may be an effective strategy to improve quality of life and reduce dyspnea in patients with Long COVID-19. The meta-analysis showed that respiratory telerehabilitation was associated with significant improvements in quality of life, decreased dyspnea, and increased respiratory and lower extremity muscle strength and functional capacity compared to the control groups receiving standard care or placebo interventions. These findings suggest that respiratory telerehabilitation may be a valuable tool for addressing persistent symptoms and improving functionality in patients with Long COVID-19.

The systematic reviews conducted to date that attempt to clarify whether telerehabilitation is an effective and safe tool for the therapeutic approach of patients with Long COVID-19 show results similar to those found in this systematic review. In general, the results demonstrate the effectiveness and safety of telerehabilitation as a therapeutic tool to improve functional capacity, quality of life, dyspnea, and lower limb strength. However, the reviews previously carried out have a low number of studies and include patients with short- and long-term sequelae. Thus, an updated review was needed of all randomized controlled studies performed to date that included only patients with Long COVID-19.

Moreover, our results are in line with the results obtained with systematic reviews performed in other chronic respiratory pathologies, such as chronic obstructive pulmonary disease [53,54]. These reviews demonstrate the effectiveness and safety of telerehabilitation in improving physical variables and patient reported outcomes.

The effectiveness of respiratory telerehabilitation can be explained by several factors. First, remote care allows patients to access rehabilitation services from the comfort of their homes, avoiding unnecessary travel and reducing the risk of exposure to other infections, especially for those with compromised immune systems [55,56]. In addition, flexible scheduling and session availability can improve adherence to rehabilitation as patients can schedule sessions at times that are convenient for them [15,57,58].

In addition, telerehabilitation offers the ability to customize interventions for each patient, which may be especially relevant given that the symptoms and needs of patients with Long COVID-19 can vary significantly. Telerehabilitation programs can be tailored to symptom severity, functional ability, and individual preferences, which can improve the effectiveness of rehabilitation [59,60].

It is important to mention that telerehabilitation for patients with Long COVID-19 comes with certain limitations and challenges. First, not all patients may be able to participate in this type of intervention due to the heterogeneity of Long COVID-19 outcomes [61,62]. Some patients may have medical conditions or disabilities that make safe participation in telerehabilitation programs difficult. Therefore, careful individualized assessment is required before implementing this approach.

The safety of respiratory telerehabilitation is also a critical factor to take into account. The findings from the meta-analysis of adverse events indicate that respiratory telerehabilitation is a safe approach with no significant difference in adverse event rates between the telerehabilitation and control groups. This is reassuring as it suggests that telerehabilitation could be a secure choice for managing patients with Long COVID-19. Nevertheless, it remains essential to consistently monitor and assess potential side effects and adverse reactions. From the ten articles included in the systematic review and meta-analysis, only six evaluated adverse events. Additionally, the adverse effects were recorded based on the patient’s self-report, but in this way, a significant amount of information could be missing because many of the adverse effects require the observation of a professional (blood pressure, skin temperature, etc.).

Another challenge to consider is access and equity in the use of telerehabilitation. While these interventions can provide significant benefits, it is crucial to ensure that all patients have equal access to these technologies. This involves addressing economic and technological barriers that could hinder access to telerehabilitation, especially in disadvantaged or resource-limited communities.

In the context of health care and health policy, the results of this review have important implications. Telerehabilitation may be an effective solution to address the growing demand for Long COVID-19-related health services. By implementing telerehabilitation programs, health systems could optimize their resources and reduce the health care burden [63,64].

It is important to note that this review has some limitations that should be considered when interpreting the results. First, the limited number of included studies and the relatively small sample sizes may limit the generalizability of the results. In addition, although efforts were made to minimize bias and heterogeneity, some differences in study designs and intervention approaches may have contributed to the variability in the results. The heterogeneity of the different interventions makes it more difficult to standardize the results; however, the sensitivity analysis performed suggested that the effect of telerehabilitation interventions was consistent, regardless of the variability of the different interventions. However, further research with robust designs and standardized protocols is needed to confirm the findings of this review and provide a solid basis for the implementation of respiratory telerehabilitation in clinical practice.

Furthermore, this review highlights the need for further research in the field of Long COVID-19 telerehabilitation. Although the results are encouraging, further studies are needed to fully understand the long-term effects of telerehabilitation in this patient population. Future research could explore more targeted and personalized approaches as well as assess the sustainability and feasibility of implementing technology-assisted rehabilitation programs on a large scale.

## 5. Conclusions

In conclusion, this systematic review and meta-analysis provide promising evidence on the efficacy and safety of respiratory telerehabilitation in the management of persistent Long COVID-19 symptoms. The results suggest that telerehabilitation can improve quality of life, reduce dyspnea, and increase respiratory and lower extremity muscle strength and functional capacity in patients with Long COVID-19. However, caution is required when interpreting the results due to the observed heterogeneity and the limited number of included studies. Further research is needed to identify the subgroups of patients who may benefit most from telerehabilitation and to develop standardized protocols to ensure the effectiveness and safety of this intervention in Long COVID-19. Despite these limitations, respiratory telerehabilitation presents itself as a promising option for improving the care and management of patients with Long COVID-19 in the digital era.

## Figures and Tables

**Table 1:** Characteristics of the included studies.

Study (Year)	Sample Size (n (% Female)) and Age (Mean)	Time since Acute Infection (Days)	Hospital Admission (%)	Length of Hospital Stay (Days)	Severity of Acute Infection	Quality Assessment Downs and Black (Risk of Bias)
Li et al. (2021) [37]	119 (66), 51	103.7	100	26.2	Severe and critical	25 (low risk)
Del Corral et al. (2022) [38]	88 (71), 46	350.8	31.8	NR	Mild, moderate, severe, and critical	27 (low risk)
McNarry et al. (2022) [39]	148 (90), 47	270	NR	NR	Mild, moderate, severe, and critical	23 (low risk)
Okan et al. (2022) [40]	52 (48), 51	86.9	100	9.5	Severe and critical	25 (low risk)
Philip et al. (2022) [41]	150 (80), 50	320	17.3	NR	Mild, moderate, and severe	24 (low risk)
Sharma et al. (2022) [42]	30 (NR), NR	NR	NR	NR	Mild	14 (high risk)
Vallier et al. (2022) [43]	17 (29), 55	140.9	76.5	11.3	Mild, moderate, severe, and critical	19 (high risk)
Kuut et al. (2023) [44]	114 (72.8), 46	187.5	11.4	NR	Mild, moderate, severe, and critical	22 (some concerns)
Rodríguez-Blanco et al. (2023) [45]	48 (54), 41	NR	0	Not applicable	Mild	21 (low risk)
Samper-Pardo et al. (2023) [46]	100 (80), 48	483.6	NR	NR	Mild, moderate, severe, and critical	22 (low risk)

Notes: NR, not reported.

**Table 2:** Characteristics of included interventions.

Study (Year)	Intervention and Comparator Group	Telerehabilitation Components	Duration, Frequency, Volume, and Intensity	Intervention Technology and Intervention Monitoring	Outcome Measures
**Li et al. (2021)** [37]	**IG:** Breathing exercises + Aerobic exercise + Strength exercises **CG:** Educational instructions	Tele-education in self-management Symptom and mood telemonitoring PA telemonitoring with personalized feedback Teleconsultation with healthcare professionals Remote decision-support systems	6 weeks 3–4 days/week 40–60 min Divided into three levels of volume Tailored to each participant through continuous feedback	Video conference Weekly monitoring in individual sessions	**Quality of life (SF-12):** IG ** > CG (*p* < 0.05) **Dyspnea (mMRC):** IG ** > CG (*p* < 0.001) **Lung function (FVC):** IG * vs. CG * (NSD) **Functional capacity (6MWT):** IG ** > CG (*p* < 0.001) **Adverse events:** IG (0) vs. CG (0)
**Del Corral et al. (2022)** [38]	**IG1:** Respiratory training (inspiratory musculature) **IG2:** Respiratory training (inspiratory/expiratory musculature) **CG1:** Sham respiratory training **CG2:** Sham respiratory training	Tele-education in self-management Teleconsultation with healthcare professionals Telecommunication with other patients	8 weeks 6 days/week 2 times/day 20 min 6 set × 10 rep 50% of MIP	Video conference Daily monitoring in group session	**Quality of life (EQ-5D):** IG1 * vs. CG1 * (NSD) IG2 * > CG2 (*p* < 0.001) **Lung function (FVC):** IG1 vs. CG1 (NSD) IG2 vs. CG2 (NSD) **Anxiety and depression (HADS):** IG1 * vs. CG1 * (NSD) IG2 * vs. CG2 (NSD) **MIP:** IG1 * > CG1 * (*p* < 0.05) IG2 * > CG2 * (*p* < 0.05) **Functional capacity (RT):** IG1 vs. CG1 (NSD) IG2 * vs. CG2 (NSD) **Lower limb strength (1STS):** IG1 * > CG1 (*p* < 0.05) IG2 * > CG2 (*p* < 0.05) **Adverse events:** IG1 (0) vs. CG1 (1) IG2 (0) vs. CG2 (0)
**McNarry et al. (2022)** [39]	**IG:** Respiratory training **CG:** Usual care	Tele-education in self-management Symptom and mood telemonitoring	8 weeks 3 days/week 20 min 6 set × 6 rep 80% of SMIP	Video conference Not monitoring	**Quality of life (K-BILD):** IG ** vs. CG (NSD) **Dyspnea (TDI):** IG > CG (*p* < 0.05) **Adverse events:** NR
**Okan et al. (2022)** [40]	**IG:** Breathing exercises **CG:** Education brochure	Tele-education in self-management Symptom and mood telemonitoring PA telemonitoring with personalized feedback Teleconsultation with healthcare professionals	5 weeks 7 days/week 3 times/day 1 set × 10 rep NR	Mobile app Weekly monitoring in individual sessions	**Quality of life (SGRQ):** IG ** > CG ** (*p* < 0.001) **Dyspnea (mMRC):** IG ** > CG * (*p* < 0.001) **Lung function (FVC):** IG ** vs. CG (*p* < 0.001) **Functional capacity (6MWT):** IG ** > CG (*p* < 0.001) **Adverse events:** IG (0) vs. CG (0)
**Philip et al. (2022)** [41]	**IG:** Breathing control and anxiety self-management exercises **CG:** Usual care	Tele-education in self-management Symptom and mood telemonitoring PA telemonitoring with personalized feedback Teleconsultation with healthcare professionals Telecommunication with other patients Remote decision-support systems	6 weeks On-demand and adapted to each participant through continuous feedback	Video conference Weekly monitoring in group sessions	**Quality of life (SF-36):** IG > CG (*p* < 0.05) **Dyspnea (MD-12):** IG vs. CG (NSD) **Anxiety and depression (GAD-7):** IG vs. CG (NSD) **Adverse events:** IG (1) vs. CG (0)
**Sharma et al. (2022)** [42]	**IG:** Breathing exercises + Aerobic exercise + Strength exercises **CG:** Usual care	PA telemonitoring with personalized feedback	6 weeks 4 days/week NR	Mobile app Not monitoring	**Dyspnea (mMRC):** IG * > CG (*p* < 0.05) **Fatigue (VAS):** IG * > CG (*p* < 0.05) **Adverse events:** IG (0) vs. CG (0)
**Vallier et al. (2022)** [43]	**IG:** Aerobic exercise + Strength exercises + Relaxation exercises **CG:** Face-to-face physiotherapy	Symptom and mood telemonitoring PA telemonitoring with personalized feedback Teleconsultation with healthcare professionals	4 weeks 4 days/week 40–60 min 90–100% HR	Video conference Weekly monitoring in individual session	**Quality of life (VQ11):** IG * vs. CG * (NSD) **Dyspnea (mMRC):** IG * vs. CG * (NSD) **Lung Function (FVC):** IG * vs. CG * (NSD) **Fatigue (MFI):** IG ** > CG ** (*p* < 0.05) **Functional capacity (6MWT):** IG ** vs. CG ** (NSD) **Lower limb strength (1STS):** IG ** vs. CG ** (NSD) **Adverse events:** NR
**Kuut et al. (2023)** [44]	**IG:** Multidisciplinary telerehabilitation based on cognitive behavioral therapy **CG:** Usual care	Tele-education in self-management Symptom and mood telemonitoring PA telemonitoring with personalized feedback Teleconsultation with healthcare professionals Telecommunication with other patients Remote decision-support systems	17 weeks On demand and tailored to each participant	Video conference On-demand monitoring in individual sessions	**Quality of life (SF-36):** IG > CG (*p* < 0.001) **Fatigue (CIS-F):** IG > CG (*p* < 0.001) **Adverse events:** IG (8) vs. CG (20)
**Rodríguez-Blanco et al. (2023)** [45]	**IG:** Breathing exercises + Strength exercises **CG:** No intervention	Tele-education in self-management Symptom and mood telemonitoring PA telemonitoring with personalized feedback Teleconsultation with healthcare professionals Remote decision-support systems	2 weeks 7 days/week 30 min 1 set × 12 rep Tailored to each participant through continuous feedback	Video conference Daily monitoring in individual session	**Dyspnea (MD-12):** IG ** > CG (*p* < 0.001) **Fatigue (VAS):** IG ** > CG (*p* < 0.001) **Functional capacity (6MWT):** IG ** > CG (*p* < 0.001) **Lower limb strength (30STS):** IG ** > CG (*p* < 0.001) **Adverse events:** IG (0) vs. CG (0)
**Samper-Pardo et al. (2023)** [46]	**IG:** Breathing exercises + Aerobic exercise **CG:** Usual care	Tele-education in self-management Symptom and mood telemonitoring PA telemonitoring with personalized feedback Teleconsultation with healthcare professionals Telecommunication with other patients	12 weeks On demand and tailored to each participant	Mobile app Not monitoring	**Quality of life (SF-36):** IG vs. CG (NSD) **Anxiety and depression (HADS):** IG vs. CG (NSD) **Lower limb strength (30STS):** IG vs. CG (NSD) **Adverse events:** NR

Notes: * Difference with respect to the baseline *p* < 0.05. ** Difference with respect to the baseline *p* < 0.001. 1STS, 1 min Sit-to-Stand Test; 30STS, 30 s Sit-to-Stand Test; 6MWT, 6 Minutes Walking Test; CG, Control group; CIS-F, Fatigue severity subscale of the Checklist Individual Strength; EQ-5D, EuroQol 5-Dimension; FVC, Forced Vital Capacity; GAD-7, Generalized Anxiety Disorder 7; HADS, Hospital Anxiety and Depression Scale; HR, Heart rate; IG, Intervention group; K-BILD, Kansas City Pulmonary–Behavioral Inventory of Lung Disease; MD-12, Multidimensional Dyspnea-12; MFI, Multidimensional Fatigue Inventory; MIP, Maximal Inspiratory Pressure; mMRC, Modified Medical Research Council; NR, Not reported; NSD, Non-Significant Difference; PA, Physical activity; RT, Ruffier Test; SF-12, Short Form 12 Health Survey; SGRQ, St. George’s Respiratory Questionnaire; SMIP, Sustained Maximal Inspiratory Pressure; TDI, Transition Dyspnea Index; VAS, Visual Analog Scale; VQ11, Short health-related quality of life questionnaire.

## Data Availability

No additional data are available.

## Appendix A. Search Strategy

Medline (via Pubmed): (“post-acute COVID-19 syndrome” OR “post-acute COVID syndrome” OR “long-COVID” OR “long COVID” OR “long-haul COVID” OR “long haul COVID” OR “persistent COVID-19” OR “long hauler COVID” OR “post-acute sequelae of SARS-CoV-2 infection” OR “chronic COVID syndrome” OR “post-COVID” OR “post COVID” OR “long-term COVID-19” OR “post-COVID syndrome” OR “post-COVID-19 syndrome” OR “post COVID-19 condition” OR “post-COVID-19 condition” OR “post-COVID-19 conditions” OR “post-COVID conditions” OR “post-COVID condition” OR “COVID-19 persistent symptoms” OR “COVID-19 consequences” OR “Ongoing symptomatic COVID-19”) AND (“Physical Therapy Modalities” OR “modalities, Physical Therapy” OR “modality, Physical Therapy” OR “physical Therapy Modality” OR “physiotherapy (Techniques)” OR “physiotherapies (Techniques)” OR “physiotherapy” OR “physical” OR “therapy” OR “therapies” OR “physical Therapy Techniques” OR “physical Therapy Technique” OR “techniques, Physical Therapy” OR “group Physiotherapy” OR “group Physiotherapies” OR “physiotherapies, Group” OR “physiotherapy, Group” OR “physical Therapy” OR “physical Therapies” OR “therapy, Physical” OR “neurological Physiotherapy” OR “physiotherapy, Neurological” OR “neurophysiotherapy” OR “Physical Therapy Specialty” OR “Specialty, Physical Therapy” OR “Therapy Specialty, Physical” OR “Physiotherapy Specialty” OR “Specialty, Physiotherapy” OR “Rehabilitation” OR “Habilitation” OR “Exercise Therapy” OR “Remedial Exercise” OR “Exercise, Remedial” OR “Exercises, Remedial” OR “Remedial Exercises” OR “Therapy, Exercise” OR “Exercise Therapies” OR “Therapies, Exercise” OR “Rehabilitation Exercise” OR “Exercise, Rehabilitation” OR “Exercises, Rehabilitation” OR “Rehabilitation Exercises” OR “activity” OR “activities” OR “exercise” OR “training” OR “Exercise Movement Techniques” OR “Movement Techniques, Exercise” OR “Exercise Movement Technics” OR “Pilates-Based Exercises” OR “Exercises, Pilates-Based” OR “Pilates Based Exercises” OR “Pilates Training” OR “Training, Pilates” OR “Telerehabilitations” OR “Tele-rehabilitation” OR “Tele rehabilitation” OR “Tele-rehabilitations” OR “Remote Rehabilitation” OR “Rehabilitation, Remote” OR “Rehabilitations, Remote” OR “Remote Rehabilitations” OR “Virtual Rehabilitation” OR “Rehabilitation, Virtual” OR “Rehabilitations, Virtual” OR “Virtual Rehabilitations”)

Scopus: TITLE-ABS-KEY ((“post-acute COVID-19 syndrome” OR “post-acute COVID syndrome” OR “long-COVID” OR “long COVID” OR “long-haul COVID” OR “long haul COVID” OR “persistent COVID-19” OR “long hauler COVID” OR “post-acute sequelae of SARS-CoV-2 infection” OR “chronic COVID syndrome” OR “post-COVID” OR “post COVID” OR “long-term COVID-19” OR “post-COVID syndrome” OR “post-COVID-19 syndrome” OR “post COVID-19 condition” OR “post-COVID-19 condition” OR “post-COVID-19 conditions” OR “post-COVID conditions” OR “post-COVID condition” OR “COVID-19 persistent symptoms” OR “COVID-19 consequences” OR “Ongoing symptomatic COVID-19”) AND (“Physical Therapy Modalities” OR “modalities, Physical Therapy” OR “modality, Physical Therapy” OR “physical Therapy Modality” OR “physiotherapy (Techniques)” OR “physiotherapies (Techniques)” OR “physiotherapy” OR “physical” OR “therapy” OR “therapies” OR “physical Therapy Techniques” OR “physical Therapy Technique” OR “techniques, Physical Therapy” OR “group Physiotherapy” OR “group Physiotherapies” OR “physiotherapies, Group” OR “physiotherapy, Group” OR “physical Therapy” OR “physical Therapies” OR “therapy, Physical” OR “neurological Physiotherapy” OR “physiotherapy, Neurological” OR “neurophysiotherapy” OR “Physical Therapy Specialty” OR “Specialty, Physical Therapy” OR “Therapy Specialty, Physical” OR “Physiotherapy Specialty” OR “Specialty, Physiotherapy” OR “Rehabilitation” OR “Habilitation” OR “Exercise Therapy” OR “Remedial Exercise” OR “Exercise, Remedial” OR “Exercises, Remedial” OR “Remedial Exercises” OR “Therapy, Exercise” OR “Exercise Therapies” OR “Therapies, Exercise” OR “Rehabilitation Exercise” OR “Exercise, Rehabilitation” OR “Exercises, Rehabilitation” OR “Rehabilitation Exercises” OR “activity” OR “activities” OR “exercise” OR “training” OR “Exercise Movement Techniques” OR “Movement Techniques, Exercise” OR “Exercise Movement Technics” OR “Pilates-Based Exercises” OR “Exercises, Pilates-Based” OR “Pilates Based Exercises” OR “Pilates Training” OR “Training, Pilates” OR “Telerehabilitations” OR “Tele-rehabilitation” OR “Tele rehabilitation” OR “Tele-rehabilitations” OR “Remote Rehabilitation” OR “Rehabilitation, Remote” OR “Rehabilitations, Remote” OR “Remote Rehabilitations” OR “Virtual Rehabilitation” OR “Rehabilitation, Virtual” OR “Rehabilitations, Virtual” OR “Virtual Rehabilitations”))
